# Development of fluorophores for the detection of oligomeric aggregates of amyloidogenic proteins found in neurodegenerative diseases

**DOI:** 10.3389/fchem.2023.1343118

**Published:** 2023-12-22

**Authors:** Kristine L. Teppang, Qilin Zhao, Jerry Yang

**Affiliations:** Department of Chemistry and Biochemistry, University of California San Diego, San Diego, CA, United States

**Keywords:** fluorescence, probes, proteins, neurodegenerative diseases, oligomers

## Abstract

Alzheimer’s disease and Parkinson’s disease are the two most common neurodegenerative diseases globally. These neurodegenerative diseases have characteristic late-stage symptoms allowing for differential diagnosis; however, they both share the presence of misfolded protein aggregates which appear years before clinical manifestation. Historically, research has focused on the detection of higher-ordered aggregates (or amyloids); however, recent evidence has shown that the oligomeric state of these protein aggregates plays a greater role in disease pathology, resulting in increased efforts to detect oligomers to aid in disease diagnosis. In this review, we summarize some of the exciting new developments towards the development of fluorescent probes that can detect oligomeric aggregates of amyloidogenic proteins present in Alzheimer’s and Parkinson’s disease patients.

## 1 Introduction

**FIGURE 1:**
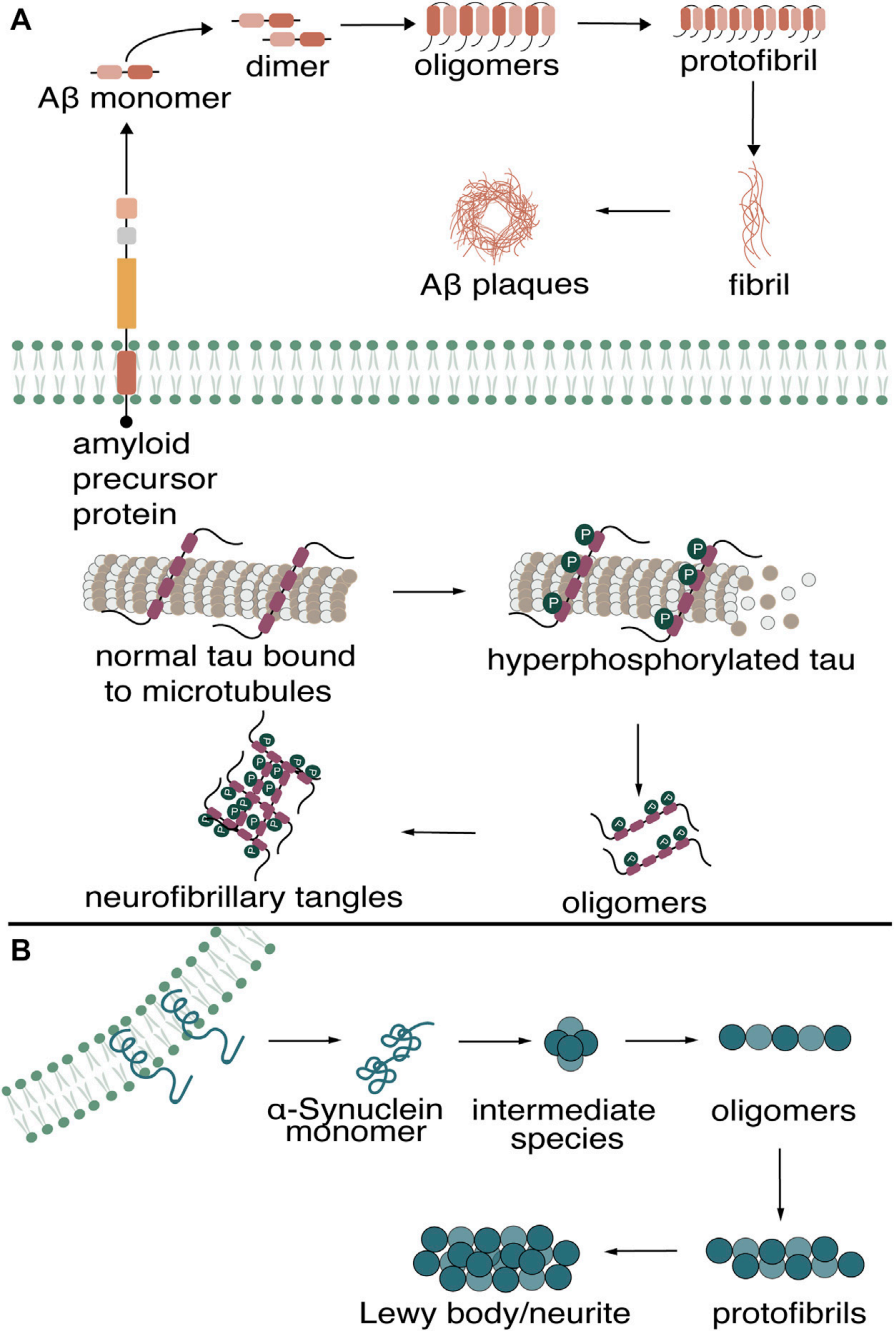
Misfolding of Aβ, tau in **(A)** Alzheimer’s Disease **(B)** and α-Synuclein in Parkinson’s Disease into higher-ordered aggregates.

The misfolding and aggregation of proteins into amyloids is characteristic of many neurodegenerative diseases (NDs). Of the NDs, the two most common globally are Alzheimer’s disease (AD) and Parkinson’s disease (PD) (Nussbaum and Ellis, 2003). AD is a progressive disease continuum that affects cognition, function, and behavior, while PD is a multi-attribute disorder that combines motor and nonmotor symptoms (Váradi, 2020; Porsteinsson et al., 2021). Diagnosis of these diseases, when a patient is already exhibiting clinical symptoms, is often too late for the efficacies of many current therapeutics. However, research has shown that the deposition of these amyloids in the brain appears years before the manifestation of clinical AD and PD symptoms; therefore, the early detection of amyloid biomarkers may aid in diagnosis at a stage where therapeutic intervention could be effective (Taylor et al., 2002). On a molecular level, patients who develop AD symptoms accumulate beta-amyloid (Aβ) aggregates and tau neurofibrillary tangles (NFTs) (Selkoe, 2001; Aisen et al., 2017; Porsteinsson et al., 2021). For PD patients, α-Synuclein is a major component underlying the Lewy body deposits associated with the disease (Meade et al., 2019). The monomeric forms of these proteins undergo misfolding, putatively resulting in a similar cascade of events that lead to various aggregation states. These monomers aggregate into soluble dimers, trimers, oligomers, and eventually into insoluble, higher-ordered aggregates rich in β-sheet content. For AD patients, the aberrant cleavage of the amyloid precursor protein results in the formation of Aβ plaques, while the hyperphosphorylation of tau precedes the formation of neurofibrillary tangles (NFTs) (Figure 1A). The balance between α-Synuclein production and clearance in PD patients is disrupted, causing monomers to aggregate into Lewy bodies/neurites (Figure 1B).

Imaging the brain and detecting protein biomarkers has become the gold standard for aiding in diagnosis of AD and PD in the clinic. Magnetic resonance imaging (MRI) of the brain allows for visualization of atrophy; however, at this stage, diagnosis is too late for current drug treatments. Amyloid-targeting radiolabeled probes visualized through positron emission tomography (PET) are presently used in the clinic to aid in diagnosing AD and PD in living patients (Ono et al., 2017; Bao et al., 2021). While PET is widely used in the clinic to detect amyloids in the brain, PET-enabling radioligands are limited by short half-lives, high production costs, and limited accessibility outside of the clinic. These limitations have resulted in an urgent need for more accessible diagnostic methods for AD and PD. The development of fluorescent molecules as a sensitive and noninvasive method for detecting proteins is widely used throughout modern research. Compared to PET, fluorescence combined with optical imaging provides an alternative approach that is more cost-effective, has improved resolution (higher spatiotemporal control), and can be used as a diagnostic tool for the detection of amyloid biomarkers in cerebrospinal fluid (CSF), urine, and the eye (Cao and Yang, 2018).

Numerous research groups have focused heavily on the detection of higher-ordered aggregates of amyloidogenic proteins. Their common high β-sheet content is a structural motif that can be targeted by multiple fluorescent dyes. However, more recent evidence has shown that the oligomeric state of these proteins is more neurotoxic, which has led to increased efforts towards the development of probes that can also detect oligomeric species (Glabe, 2008; Larson and Lesné, 2012; Kayed and Lasagna-Reeves, 2013; Verma et al., 2015). While numerous fluorescent probes have been developed to detect highly aggregated species, detecting these oligomeric species by fluorescence has proven more challenging. The difficulty in detecting these oligomers can be attributed to several factors: 1) heterogeneity and metastability of oligomers, 2) oligomers and higher-ordered aggregates sharing the same primary amino acid sequence, and 3) the lack of structural information of oligomers that enables reliable design of small molecules for targeting oligomers (Lo et al., 2019; Pilkington and Legleiter, 2019; Lo, 2022; Shea and Daggett, 2022; Vaikath et al., 2022; Wang et al., 2023).

In this review, we will discuss efforts reported in the literature towards the development of fluorescent probes that can detect oligomeric species of Aβ, tau, and α-Synuclein. We will highlight potential design guidelines for advancing fluorophores with improved specificity for oligomers. A survey of literature emphasizes the urgent need for fluorescent probes with oligomer specificity. This review will discuss fluorescent probes that bind to various aggregation states, including oligomers. The strategies for detecting oligomers varies depending on the target protein; therefore, we will summarize tools that multiple researchers have used for each protein used to generate oligomers individually.

## 2 Fluorescent probes that detect aggregated amyloidogenic species found in Alzheimer’s disease

Much of AD research towards the development of diagnostic agents has focused on detecting amyloid deposits comprised primarily of insoluble Aβ plaques and tau NFTs. These higher-ordered aggregates possess β-sheet-rich character, allowing for a common structural motif that small molecule fluorescent probes can target. We will refer the reader to other reviews for a comprehensive discussion of fluorescent probes reported to detect higher-ordered amyloid deposits of Aβ and tau (Nilsson, 2009; Reinke and Gestwicki, 2011; Tong et al., 2015; Su et al., 2022).

Although the deposition of insoluble Aβ plaques and tau NFTs in the brain serve as pathological hallmarks in AD, recent evidence of the detrimental role of oligomers and the lack of accessible and reliable chemical tools for their detection has resulted in increased recent efforts towards developing fluorescent probes to detect these soluble, toxic oligomeric species. Many fluorescent probes that detect these higher-ordered aggregates show no binding capability to oligomers. The development of fluorescent probes that can detect oligomeric species of Aβ has proven to be a more challenging and largely underdeveloped area of research. Furthermore, progress in developing an oligomer-specific fluorescent probe for tau is even more limited.

### 2.1 The development of fluorescent probes that target Aβ oligomers

**FIGURE 2:**
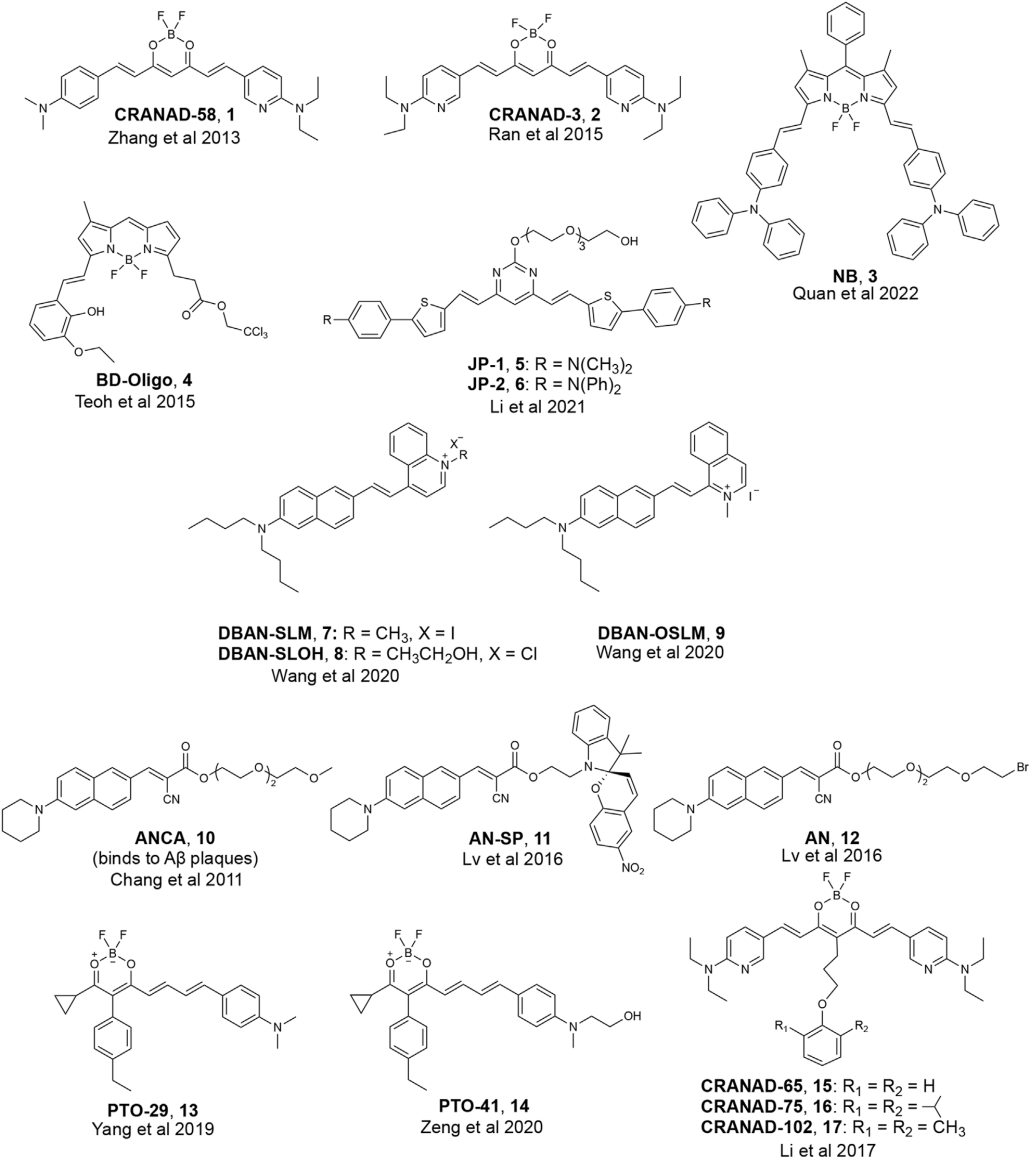
Examples of fluorescent probes reported to detect Aβ oligomers.

Of the three proteins that form amyloid deposits in AD and PD (Aβ, tau, α-Synuclein), more reported efforts have been made towards the development of fluorescent probes for Aβ oligomers, which can be partially attributed to the increased availability of structural models for varying Aβ species. We will first highlight the development of fluorescent probes that can detect all aggregated Aβ species, including oligomers. Many reports on these probes have speculated on potential binding motifs that may allow for targeting Aβ oligomers. Structures of various fluorescent probes that have been reported to detect Aβ oligomers are presented in Figure 2.

**FIGURE 3:**
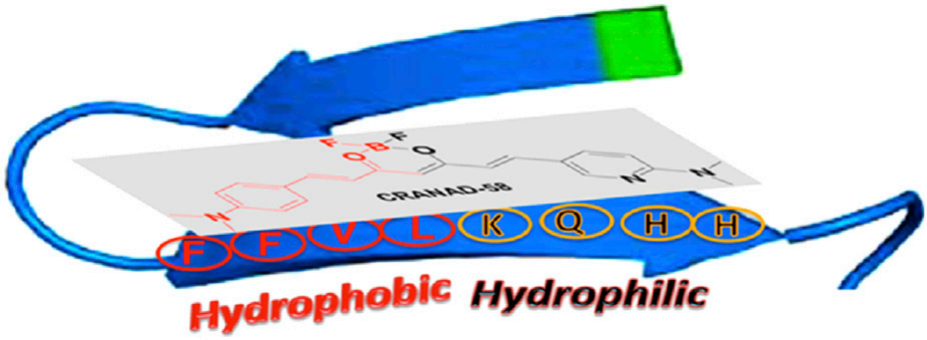
Proposed complex between **CRANAD-58** and an Aβ peptide monomer. Adapted with permission from (Zhang et al., 2013). Copyright 2013 American Chemical Society.

Most researchers who have reported on detecting soluble, oligomeric Aβ have employed computer-aided analysis to understand how their fluorescent probes can detect soluble Aβ species. Initial design principles for binding to Aβ oligomers, for instance, were based on structural analysis of fragments of monomeric Aβ. For example, Ran and coworkers hypothesized that interactions with the HHQKLVFF segment on the Aβ peptide would allow for binding to all forms of Aβ, including the more toxic oligomeric species. The HHQK fragment is a hydrophilic segment representative of soluble Aβ species, and the LVFF segment is a more sterically hindered hydrophobic segment. The authors reported on the ability of curcumin analogs, **CRANAD-58** and **CRANAD-3,** to bind to all Aβ species in solution and in the brain of a transgenic mouse model (Zhang et al., 2013; Zhang et al., 2015). Molecular docking studies of **CRANAD-58** suggest that the hydrophilic segment of the probe interacts with the hydrophilic part of Aβ allowing for the detection of oligomers, and the hydrophobic moiety of the probe interacts with the hydrophobic components of Aβ allowing for the simultaneous detection of aggregates (Figure 3). Focusing on interactions with an even smaller segment of Aβ, the Wu lab synthesized BF2-dipyrrolmethane fluorescence-imaging probe (**NB**) (Figure 2) that can detect different aggregation states of Aβ (Quan et al., 2023). The design of **NB** was based on the detection of diphenylalanine (FF), the smallest unit and core recognition motif of Aβ that plays a crucial role in AD pathogenesis (Görbitz, 2006; Ji et al., 2021).

**FIGURE 4:**
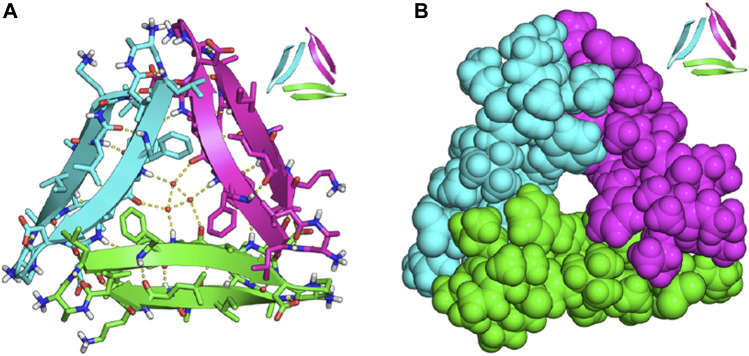
X-ray crystallographic structure of trimeric PDB model 4NTR of Aβ_17-36_ **(A)** trimeric peptide structure with ordered water molecules located in the internal cavity; **(B)** the same trimeric structure as in **(A)**, but in space-filling representation to highlight the internal cavity that may be targeted by small molecules. Adapted with permission from (Spencer et al., 2014). Copyright ACS AuthorChoice License.

The emergence of a trimeric Aβ model, Protein Data Bank (PDB) model 4NTR by the Nowick lab (which will be referred to as PDB 4NTR in the remainder of this review), significantly paved the way for developing fluorescent probes with improved specificity to Aβ oligomers (Spencer et al., 2014). A survey of the literature shows that many research groups utilized this model in their computational studies to rationalize the ability of their probes to detect oligomers. This structure is based on a synthetic peptide derived from Aβ_17-36_ that crystallizes to form trimers and further aggregates to form other oligomers. This structure contains three β-hairpins that assemble triangularly and interlock to create a trimer, with each β-hairpin making up one side of the pseudo-equilateral triangle (Figure 4). In the center of this pseudo-equilateral triangle appears to be a potential binding pocket for small molecules with hydrophobic F19/V36 residues exposed to solvents. These residues are exclusively exposed to solvent in oligomers and are not present in Aβ fibrils; therefore, several groups have suggested that interaction with these hydrophobic residues can potentially be used to differentially target Aβ oligomers from Aβ fibrils (Lee and Ham, 2011; Teoh et al., 2015; Lv et al., 2016).

Due to the lack of clear, rational design principles for the development of oligomeric-specific Aβ probes, the Chang lab generated a diversity-oriented fluorescence library and performed high-throughput screening of 3,500 compounds (Teoh et al., 2015) to screen for compounds that bound to Aβ oligomers. This study resulted in the identification of **BD-Oligo** (Figure 2), which exhibited a fluorescence increase at early time points in an Aβ aggregation assay (where it is expected that oligomeric Aβ species will be most prevalent in solution) and a decrease in fluorescence signal at later time points when the solution was expected to contain a high fraction of amyloid fibrils. Transmission electron microscopy (TEM) experiments support the hypothesis that the fluorescence increase could be due to the binding of **BD-Oligo** to oligomeric species that formed at early time points in this assay. In contrast, the fluorescence decline of **BD-Oligo** followed the disappearance of oligomers and the formation of fibrils in the sample. Molecular docking studies with **BD-Oligo** in PDB 4NTR suggest that **BD-Oligo** adopts a conformational transition from a planar to twisted geometry to increase interactions with the Aβ trimer. In addition, docking studies indicated that the BODIPY ring and phenyl ring interact with F19/V36 (hydrophobic) residues, suggesting these interactions may play a dominant role in the specificity of **BD-Oligo** to recognize trimers (Lee and Ham, 2011). Despite showing promise for oligomer specificity in solutions containing synthetic Aβ, **BD-Oligo** was capable of staining Aβ plaques in brain slices from mature 18-month-old APP/PS1 mice, which presumably have mostly fibrillar species. It, therefore, remains to be seen whether **BD-Oligo** will exhibit the specificity for Aβ oligomers that could reveal new information than what is currently possible in a clinical setting.

**FIGURE 5:**
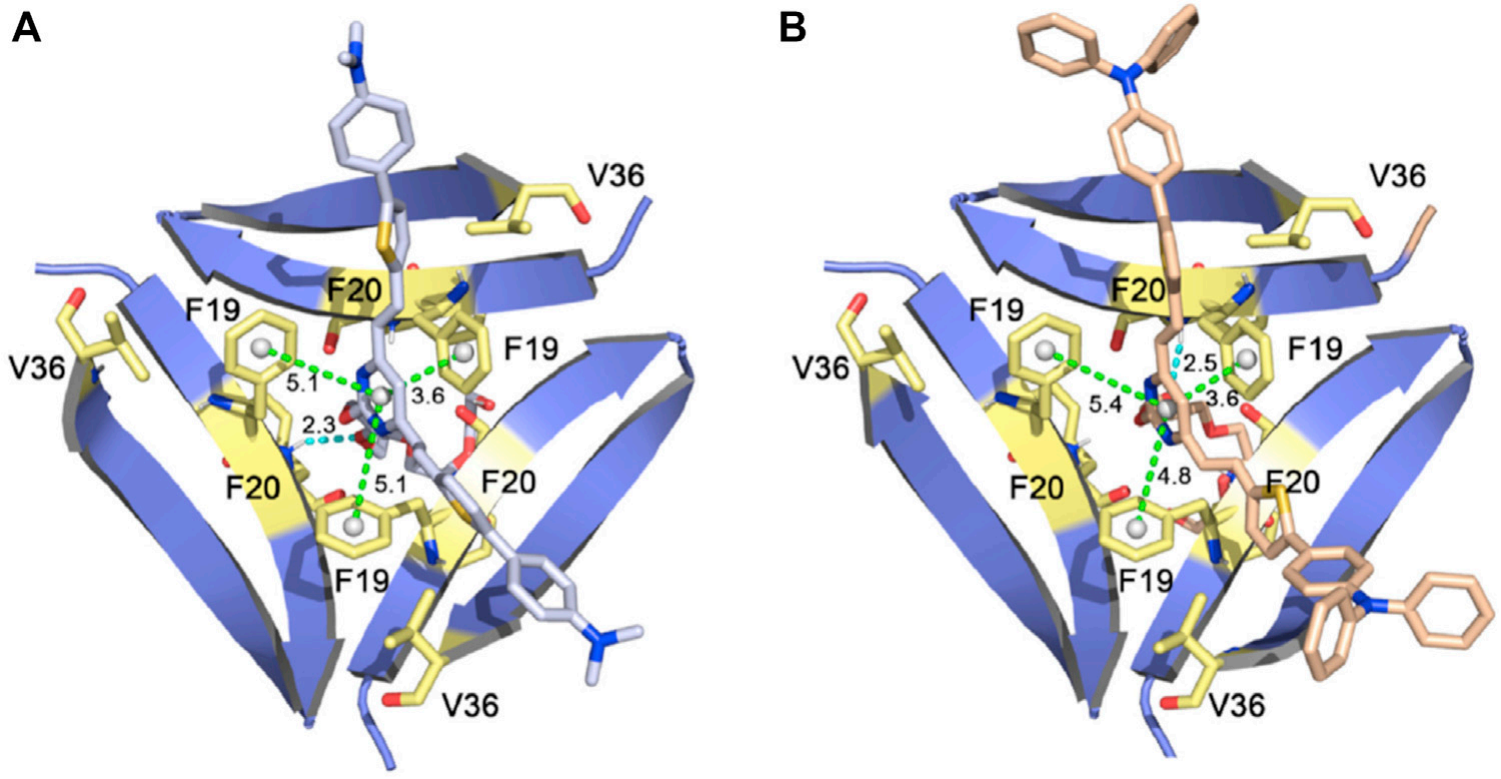
**(A) JP-1** and **(B) JP-2** docked into PDB 4NTR. Adapted with permission from (Li et al., 2021). Copyright from 2021 Elsevier.

In another study, the Pan lab developed **JP-1** and **JP-2**, two curcuminoid derivatives (Figure 2) (Li et al., 2021). Molecular docking studies with PDB 4NTR suggest that interaction with Aβ trimers was due to π-π stacking interactions between the pyrimidine moieties and the benzene rings of three F19 residues in the binding pocket of Aβ trimers (Figure 5). Solution-based studies with synthetic Aβ and staining in a transgenic mouse model confirm the ability of **JP-1** and **JP-2** to detect all species of Aβ. Interestingly, the authors also observed the ability of **JP-1** and **JP-2** to inhibit fibril growth, which was confirmed with TEM.

In addition to studying hydrogen-bonding and π-π stacking interactions between probes and F19/V36 residues of PDB 4NTR, another related strategy for designing fluorescent probes that bind to Aβ oligomers is by also taking advantage of probe accessibility and steric hindrance of the Aβ oligomer binding pocket. Many groups hypothesized that creating fluorescent probes with steric bulk would trap or increase probe interactions with the central cavity/binding pocket of Aβ oligomers.

The Li and Wong labs published a study investigating three naphthylamine-based cyanines: **DBAN-SLM**, **DBAN-SLOH**, and **DBAN-OSLM** (Figure 2) (Wang et al., 2021). Of the three probes, **DBAN-SLM** showed selective fluorescent responses to Aβ monomers and oligomers. Molecular modeling studies with PDB 4NTR suggest interactions between the quinoline moiety of **DBAN-SLM** and hydrophobic F19/V36 residues. In addition, molecular docking supports that **DBAN-SLM** conforms to a slightly twisted geometry with the quinolinium ring freely entrapping in the hydrophobic pocket.

Previously, the Yang lab reported on a family of fluorescent probes with an amino naphthalenyl-2-cyanoacrylate (**ANCA**) motif specific for higher ordered Aβ aggregates (Chang et al., 2011). Drawing inspiration from **ANCA** and their previous work on spiropyran (SP) derivatives, the Sun and Yi labs designed **AN-SP,** which incorporated an SP moiety into the **ANCA** scaffold (Lv et al., 2016). **AN-SP** showed the most considerable fluorescence enhancement with Aβ oligomers, and very little fluorescence enhancement was observed in the presence of Aβ fibrils, amylin fibrils (an aggregation-prone peptide secreted with insulin), and prion fibrils (another amyloid associated with prion disease) (Luca et al., 2007; Colby and Prusiner, 2011). The authors hypothesized that this specificity towards oligomers might be due to the SP moiety providing rigidity and steric hindrance, precluding binding to fibrillar binding pockets. To test this hypothesis, the authors also synthesized **AN**, which replaced the SP moiety with a flexible chain, and found that **AN** showed no specificity between Aβ oligomers and fibrils. Molecular docking of **AN-SP** to PDB 4NTR suggested that the SP unit intercalates with the hydrophobic region of the Aβ trimer. Staining in a transgenic mouse line showed the ability of **AN-SP** to stain Aβ oligomers in the brain.

Similarly, the Li group reported on **PTO-29** (Figure 2), which exhibited high selectivity to Aβ oligomers (Yang et al., 2020). The authors described the binding pocket of PDB 4NTR as “v-shaped” and hypothesized that a “v-shaped” wedge probe would allow for increased interactions with F19/V36 residues. Molecular docking studies of **PTO-29** suggest that the phenyl rings of **PTO-29** stacked between F19 and the N, N-dimethylbenzene inserts into the protein cavity, which suggests tighter binding affinity to trimers. To improve the biocompatibility of **PTO-29**, the same group modified **PTO-29** to include a hydroxyethyl group as in **PTO-41**. Computational studies with PDB 4NTR suggested that the hydroxyethyl group is inserted into the binding pocket, presumably leading to increased affinity towards trimers compared to probes that lacked this hydroxyethyl group. *In vivo* imaging of **PTO-41** in a 4-month transgenic mouse, presumably containing a higher proportion of Aβ oligomers, suggests that **PTO-41** can detect Aβ oligomers.

**FIGURE 6:**
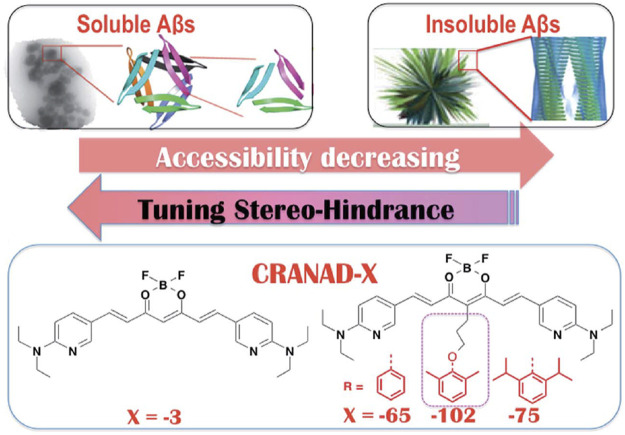
Proposed increase in stereo-hindrance of **CRANAD** analogs is accompanied by selectivity towards Aβ oligomers over insoluble Aβ species. Adapted with permission from (Li et al., 2017). Copyright 2017 shared under CC BY 3.0.

In addition to studying interactions between probes and F19/V36 residues of the Aβ trimer, another strategy employed by researchers involved taking advantage of probe accessibility and differences in binding pocket sizes of Aβ species. A comparison of the binding pockets of Aβ oligomers (PDB 4NTR) with Aβ fibril models (PDB 2LMN) suggests that the size of the binding pocket for higher-ordered aggregates is narrower compared to the binding pocket of oligomers. For example, the Ran lab tuned for stereo-hindrance by introducing bulky functional groups that would prevent binding to the more sterically hindered Aβ fibrils and allow for the detection of oligomers. Building off previously reported **CRANAD-3**, the authors synthesized **CRANAD-65**, **CRANAD-102**, and **CRANAD-75,** presented in order of increased steric bulk, to test this strategy (Li et al., 2017) (Figure 6). The authors incorporated various phenoxy-alkyl chains at the 4-position to introduce steric bulk. **CRANAD-75** showed no fluorescence enhancement with oligomers, and the authors hypothesize that the two isopropyl groups on the phenyl ring of **CRANAD-75** were too large to access the β-sheets of the oligomers. Therefore, with **CRANAD-102,** the authors replaced the isopropyl groups in **CRANAD-75** with methyl groups and showed a higher affinity for monomers, dimers, and oligomers than higher ordered Aβ species in solution.

### 2.2 Summary of current efforts to develop Aβ oligomeric fluorescent probes

While some progress has been made toward developing fluorescent probes that can detect oligomeric species of Aβ, fluorescent probes with improved specificity and biological properties are still needed. In addition, currently there is no clear identification of molecular determinants that dictate the binding of probes to Aβ oligomers. Recent developments suggest the utility of computer-aided design of molecules for improved predictability for fluorescent probes that preferentially bind to soluble Aβ species. While some research groups have focused on targeting fragments of the Aβ peptide sequence, studies suggest that using this approach is limiting for the development of oligomer-specific probes as these fragments are present in all Aβ species. While PDB model 4NTR has shown promise as a valuable structural model for developing probes that bind to oligomeric Aβ, we caution the readers that structures based on synthetic peptides or recombinantly expressed amyloids have been observed to produce different structures from those reported in patient-derived samples (Kollmer et al., 2019; Varshavskaya et al., 2022). Many research groups have highlighted the importance of probe interactions with F19/V36 on this structural model as these residues are not exposed in fibrils, allowing for potential specificity. In addition, groups have also implemented strategies based on steric hindrance/bulk and show that the increased size of the oligomer binding pocket compared to the binding pocket in Aβ fibrils can play a useful role in the potential design of an oligomer-specific probe.

## 3 The development of fluorescent probes that target tau oligomers

**FIGURE 7:**
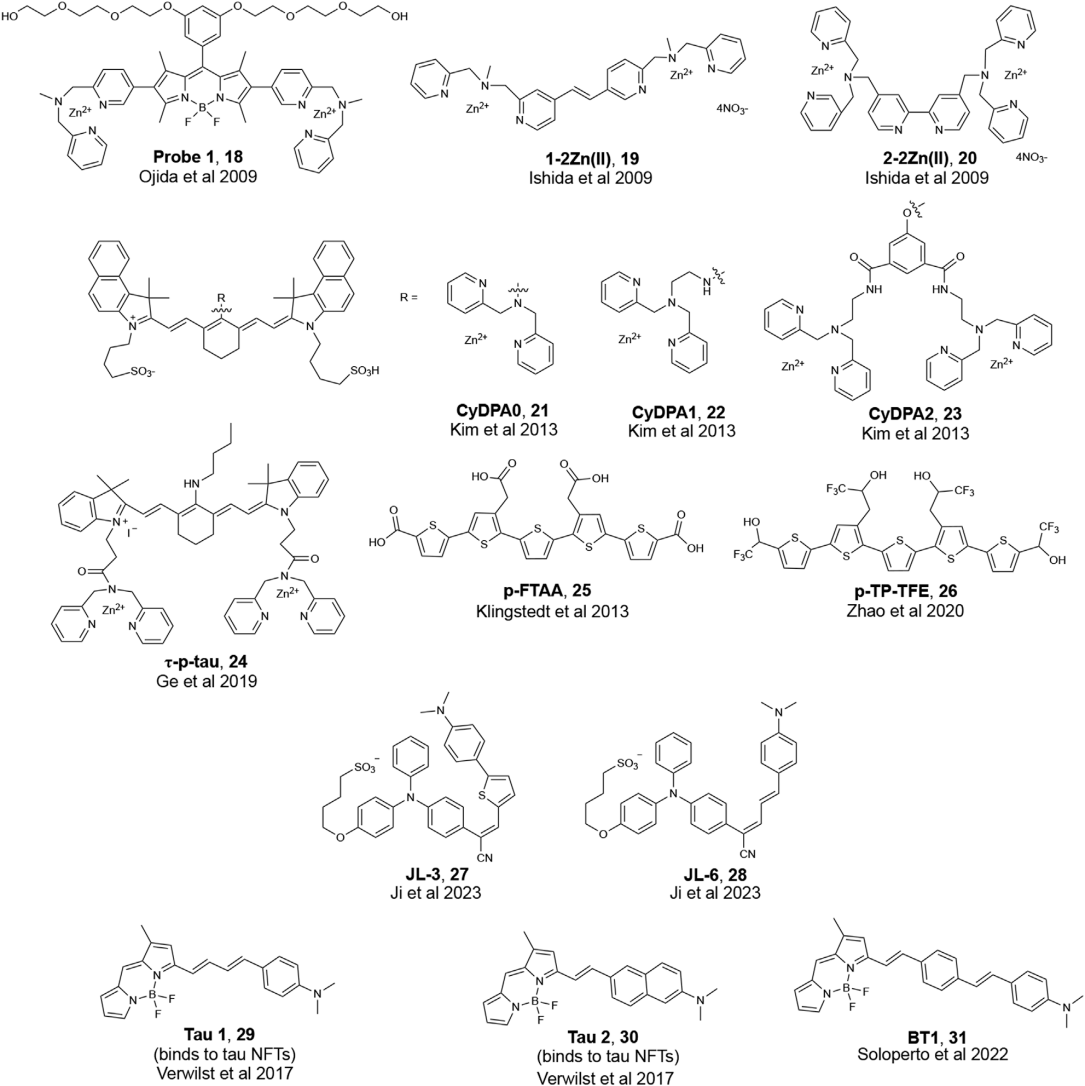
Examples of fluorescent probes that are reported to bind to phosphorylated tau.

With its earlier identification in AD patients, research in biomarker detection for AD has historically focused on the detection of amyloid plaques comprised mainly of Aβ peptides. However, with recent evidence that tau accumulation and aggregation better correlates with disease progression over Aβ, there has been increased attention towards developing methods to detect pathological tau species (Mohorko and Bresjana, 2008; Iqbal et al., 2010; Sexton et al., 2022). While it remains to be seen if this observation is universal for all scaffolds, a current hypothesis in developing fluorescent probes for tau is that increasing the fluorogenic core length of the π-network of Aβ probes leads to binding to tau aggregates (Maruyama et al., 2013; Verwilst et al., 2017). A previous review provides a detailed discussion of fluorescent probes that can bind to highly aggregated tau in neurofibrillary tangles (NFTs) (Verwilst et al., 2018). The development of fluorescent probes that bind to NFTs remains limited, and the development of tau probes that bind to phosphorylated tau oligomers is even more scarce. Given the reports on the toxicity of soluble, phosphorylated oligomeric tau species, there is an urgent need to develop fluorescent probes that can detect tau oligomers. The structure of fluorescent probes that have been reported to detect tau oligomers are presented in Figure 7. The two main strategies for the targeting of tau species include: 1) the binding of phosphorylation sites using a zinc (Zn^2+^) recognition motif, and 2) chemically modifying fluorescent probes previously reported to detect tau aggregates in NFTs. While the utility of computational studies was common for the development of probes that can detect oligomeric Aβ, the use of molecular docking to published structures of tau species is limited for the development of probes that can bind to tau oligomers.

### 3.1 Fluorescent probes that detect phosphorylation on tau proteins using a zinc (Zn^2+^) recognition motif

Evidence in literature suggests that zinc contributes to tau toxicity by increasing tau phosphorylation and directly binding to tau (Boom et al., 2009; Sun et al., 2012; Huang et al., 2014; Fichou et al., 2019). Therefore, one approach that many research groups have attempted for detecting phosphorylated tau oligomers is to use a Zn^2+^ recognition motif.

The Hamachi group, for instance, reported the detection of soluble tau oligomers by developing fluorescent probes that targeted protein hyperphosphorylation (Ishida et al., 2009; Ojida et al., 2009). Ojida et al. reported on **Probe 1** (Figure 7) with a BODIPY core and two Zn(II)-2,2′-dipicolyamine (DPA) complexes that serve as a binding site for phosphorylated amino acid residues. DPA contains nitrogen atoms that can coordinate with Zn^2+^ to form a chelate resulting in a change in the fluorescence properties of DPA probes. **Probe 1** showed a high sensitivity for hyperphosphorylated full-length tau over non-phosphorylated tau and Aβ fibrils in solution. Histological staining showed that **Probe 1** can bind to NFTs in brain sections from a confirmed AD patient; however, further studies are needed to test if **Probe 1** can also detect phosphorylated tau oligomers in tissue. Ishida et al. later developed diazastilbene analogs **1-2Zn(II)** and **2-2Zn(II)** (Figure 7), which showed a fluorescence change when bound to phosphorylated tau peptides in solution. However, studies with **1-2Zn(II)** and **2-2Zn(II)** were limited to *in vitro* examination; therefore, it remains to be seen whether these probes can label phosphorylated tau oligomers in biological samples.

The Bai lab reported on **CyDPA0**, **CyDPA1**, and **CyDPA2** (Figure 7). These probes have one or two DPA-Zn(II) complexes and are designed based on indocyanine green, a near infra-red dye (Kim et al., 2013). Of the three probes, **CyDPA2** showed the best affinity to recombinant phosphorylated tau. In gel staining of **CyDPA2** with homogenized samples from an AD patient, a transgenic mouse that overexpresses tau, and recombinant phosphorylated and non-phosphorylated tau proteins supported the ability of this probe to detect hyperphosphorylated tau oligomers (Ramsden et al., 2005; Guerrero et al., 2009). *Ex vivo* staining of brain homogenates from a tau transgenic mouse and a confirmed AD patient with **CyDPA2** supported fluorescence signals corresponding to only tau species and showed a reduction in fluorescent signals with the addition of pyrophosphate (ppi), a phosphate inhibitor.

**FIGURE 8:**
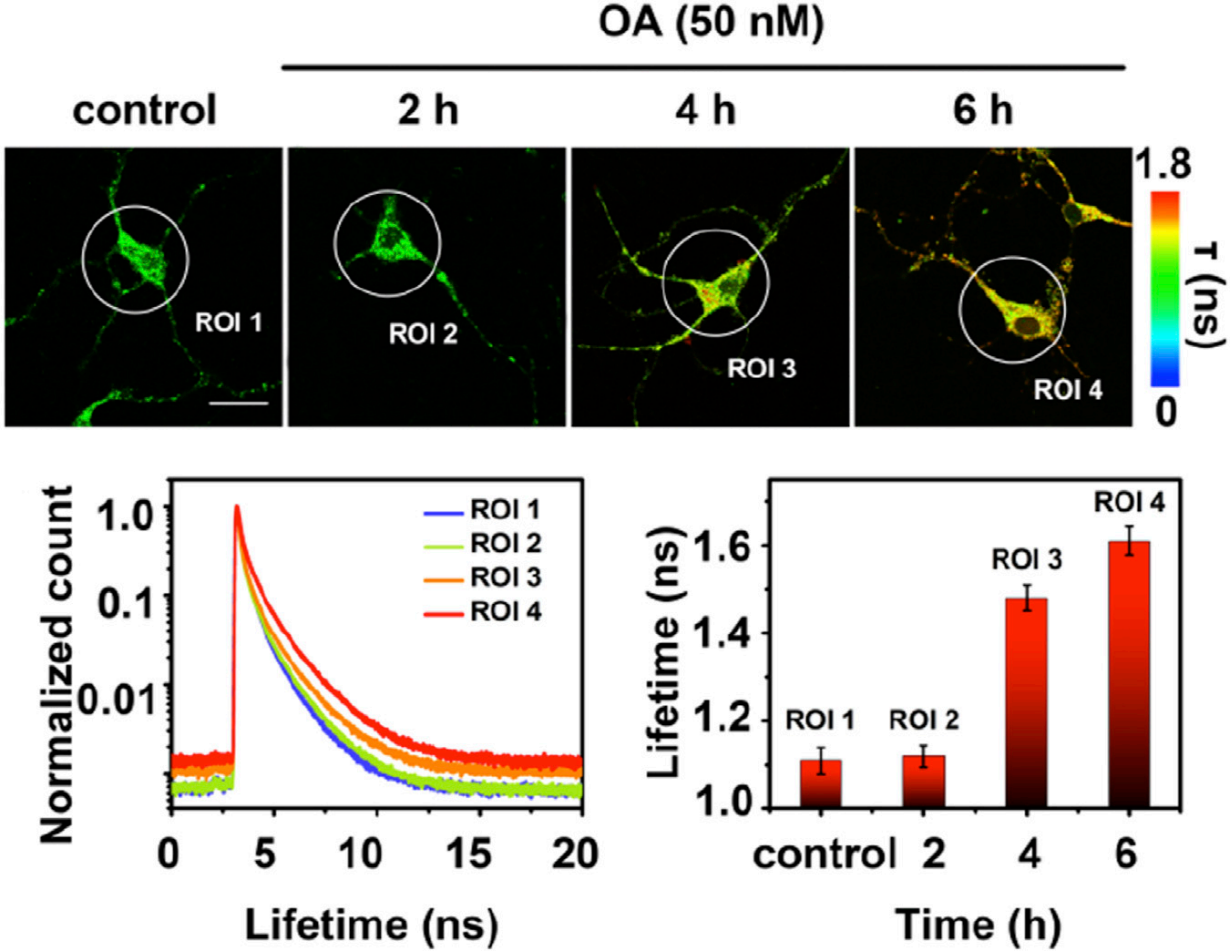
Fluorescence lifetime imaging (FLIM) of a single neuron. Neurons from a mouse model were preincubated with 50 nM Okadaic Acid (OA) to induce hyperphosphorylation prior to incubation with **τ-p-tau**. The longer lifetime (ns) of **τ-p-tau** with increased incubation of OA suggests that this probe can distinguish hyperphosphorylated tau from non-phosphorylated tau. Scale bar = 25 μm. The lifetime decay curves with the increase of hyperphosphorylation reveal their respective average fluorescence lifetime for the selected areas (ROI 1–4). Adapted with permission from (Ge and Tian, 2019). Copyright 2019 American Chemical Society.

Fluorescence lifetime imaging (FLIM) has gained increasing attention as a new microscopy technique used in biological research. When a fluorophore is excited with light, there is a time delay before these probes relax to the ground state and release a photon and this time delay is called the fluorescence lifetime (τ). FLIM images of phosphorylated tau by a cyanine probe (**τ-p-tau**, Figure 7) was reported by the Tian lab (Ge and Tian, 2019). Similar to the previous groups, the authors used two DPA units to recognize Zn^2+^ ions. **τ-p-tau** was shown to be selective for phosphorylated tau over solutions of monomeric tau, Aβ monomer, and Aβ fibril. FLIM imaging of a single neuron from a mouse model showed that **τ-p-tau** was able to monitor (via changes in lifetime) the increase in the concentration of phosphorylated tau in live cells that were pre-incubated with Okadaic acid (OA), which induces hyperphosphorylation (Figure 8). However, evidence that this probe can detect tau oligomers specifically was not reported.

### 3.2 Development of tau oligomer-responsive fluorescent probes through modification of NFT-binding dyes

**FIGURE 9:**
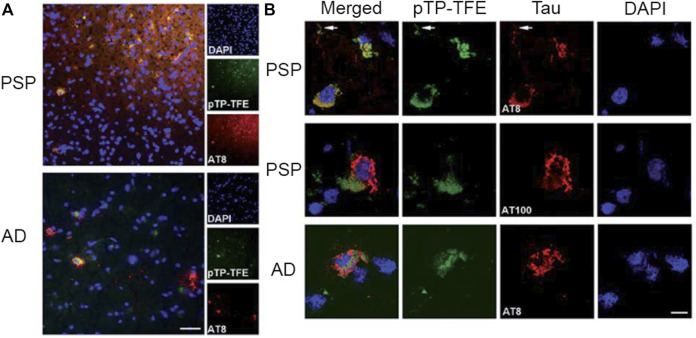
Staining of **pTP-TFE** in human progressive supranuclear palsy (PSP) and Alzheimer’s Disease (AD) brain tissue. **(A)** Representative images of **pTP-TFE** staining of PSP and AD human brain sections show overlap with an antibody (AT8) that detects phosphorylation at Ser202 and Thr205. Scale bar = 50 µm. **(B)** Confocal images of **pTP-TFE** in a PSP and an AD brain slice showing overlap with a phosphorylated tau-specific antibody (AT8) and poor overlap with filamentous tau antibody (AT100). Adapted with permission from (Y. Zhao et al., 2020). Copyright 2020 shared under CC BY-NC 3.0.

In addition to attempts at targeting tau oligomers using a Zn^2+^ recognition motif, another design strategy involves modifying fluorescent probes previously reported to bind to NFTs. The Nilsson lab reported on a series of oligothiophene-based fluorescent ligands that bind to Aβ and tau (Klingstedt et al., 2013). These analogs were designed with minor variations in their chemical structure to restrict or increase the conformation flexibility of the conjugated backbone to allow for the detection of a variety of amyloids. Of the probes reported, **pFTAA** (Figure 7) detected both NFTs and Aβ aggregates in AD patient samples. While **pFTAA** did not exhibit any apparent selectivity for various aggregate forms of Aβ and tau (soluble or insoluble), the Aigbirhio lab drew inspiration from **pFTAA** and made chemical modifications to develop pentathiophene-trifluoroethanol (**pTP-TFE**, Figure 7), a fluorescent probe that showed selectivity towards soluble, aggregated tau (Y. Zhao et al., 2020). The authors hypothesized that anionic dyes such as **pFTAA** interact with the positively charged flexible polyelectrolyte brush, or fuzzy coat, that is found on mature tau fibrils. Therefore, to minimize interactions with tau fibrils, the authors replaced the carboxylic acids on **pFTAA** with 2,2,2,-trifluoroethan-1-ol groups to generate **pTP-TFE**. **pTP-TFE** showed preferential binding to early soluble tau aggregates in solution, which was supported by TEM imaging experiments. The authors then tested the ability of **pTP-TFE** to bind to soluble tau in a confirmed AD patient and a confirmed patient with progressive supranuclear palsy (PSP), another ND that involves tau, and found that **pTP-TFE** colocalized with an antibody that detects phosphorylated tau in both patients (Figure 9).

The Yu lab designed tau-selective and aggregation-induced-emission probes, **JL-3** and **JL-6** (Figure 7) (Ji et al., 2023). **JL-3** and **JL-6** have a triphenylamine (TPA) electron-donating unit conjugated with -CN and N, N′-dimethylaminobenzene groups to form a Donor-Acceptor-Donor scaffold. Both probes showed the ability to discriminate between tau and Aβ aggregates; however, **JL-3** and **JL-6** are not oligomer-specific and bind to tau fibrils as well.

While universal rational design principles for targeting tau NFTS do not currently exist, the Kim lab showed that extending the π-network of Aβ probes enabled binding to tau NFTs (Verwilst et al., 2017). **Tau 1** and **Tau 2** (Figure 7) showed preferential binding to tau NFTs over Aβ fibrils in a transgenic mouse model. Molecular docking studies suggested that these probes interacted with amino acid residues on tau along the crystallographic structure of hexapeptide PHF6 fragment ^306^VQIVYK^311^ (Fitzpatrick et al., 2017). This hexapeptide was identified as responsible for nucleation of tau and was used in computational studies to study binding to NFTs. To target phosphorylated tau, the Boffi lab drew inspiration from **Tau 1** and further extended the π conjugation on position 3 of the BODIPY core to synthesize a family of fluorescent probes (Figure 7) (Soloperto et al., 2022). Docking to a published structure of aggregated PHF6 fragment showed that all probes interacted with tau and were docked into the central cavity of the binding site, suggesting a preference for lipophilic regions of tau. Of the probes that were designed, **BT1** showed the ability to bind to hyperphosphorylated and oligomeric tau in OA-treated human-induced Pluripotent Stem cells (hIPSC) derived neurons.

### 3.3 Summary of current efforts to develop oligomeric tau binding fluorescent probes

This section discusses recent progress and attempts at the rational design of probes that detect various tau species, including hyperphosphorylated tau oligomers. A survey of the literature suggests that there are still no clear molecular features that can be used for designing probes that specifically target soluble tau oligomers. However, one common approach for developing probes that can detect tau oligomers includes a Zn^2+^ recognition motif such as DPA, which allows for the detection of all tau species, as non-pathological tau and NFTs are also phosphorylated to a various extent. Another strategy for designing oligomeric tau targeting fluorescent probes involves making chemical modifications on probes previously reported to target NFTs; however, to the best of our knowledge, the specific detection of oligomeric species of tau in tissue has yet to be achieved and it remains to be seen whether these probes will show specificity in tissue. As with developing probes that target Aβ oligomers, we hypothesize that incorporating computer-aided design would allow researchers to pre-screen probes *in silico*, which could help advance the field in developing tau oligomer-specific fluorescent probes. While protein models for higher-ordered tau aggregates from AD patients exist, a protein model of hyperphosphorylated tau oligomers has not yet been reported (Fitzpatrick et al., 2017). A model for phosphorylated tau oligomers would be of great utility; however, until one is available, a pre-screening of molecules that can bind to the published structure of aggregated PHF6 fragment of tau may represent a reasonable starting point.

## 4 α-Synuclein and its significance in Parkinson’s disease

**FIGURE 10:**
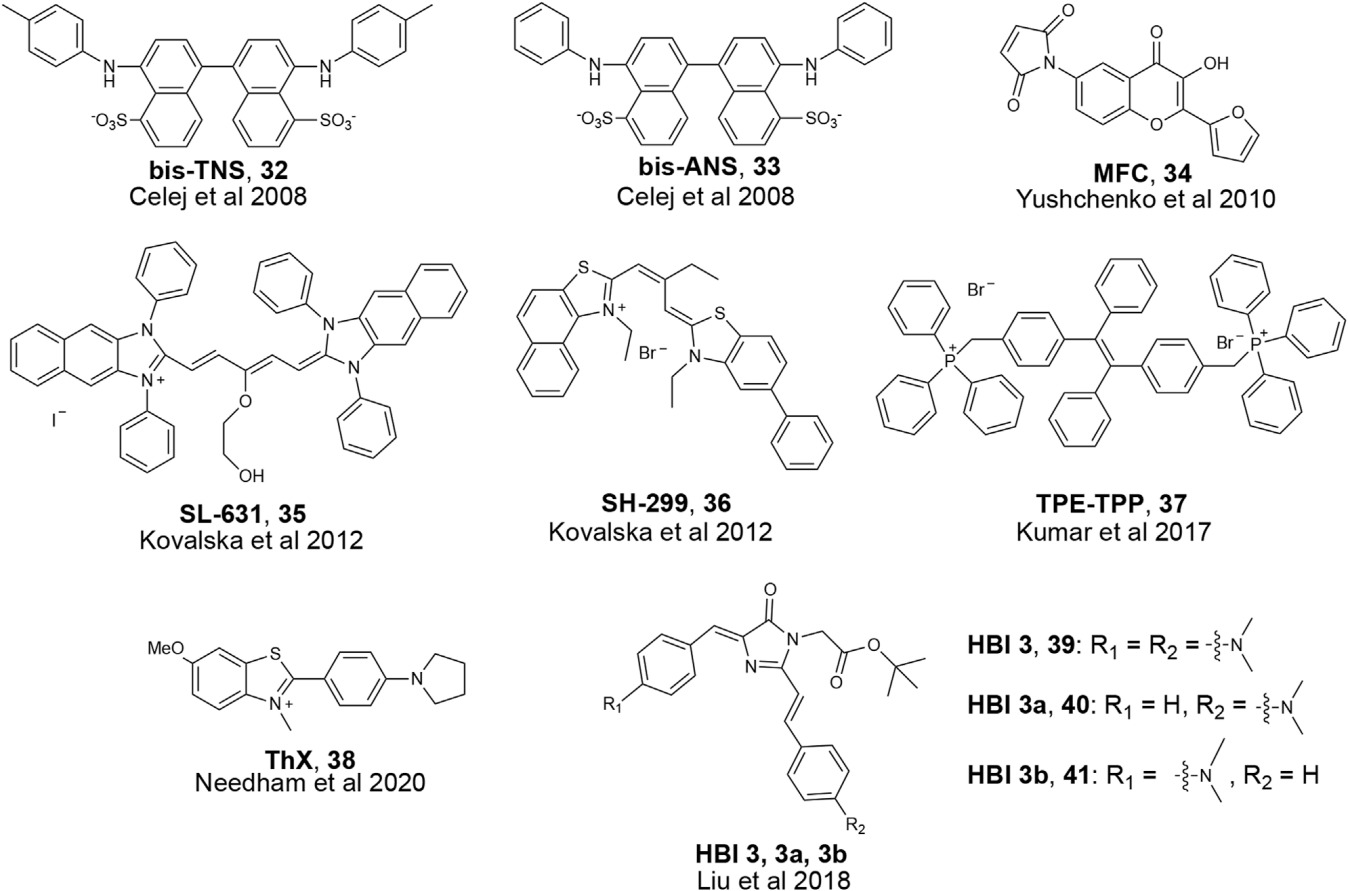
Examples of fluorescent probes reported to detect α-Synuclein oligomers.

α-Synuclein is another amyloidogenic protein that has gathered strong research interests due to its association with Parkinson’s Disease (PD) (Chiti and Dobson, 2006). In PD patients, α-Synuclein monomers aggregate to form soluble oligomers, then β-sheet rich fibrils, and eventually large insoluble deposits known as Lewy bodies and Lewy neurites based on their circular and fibril structure (Spillantini et al., 1997; Baba et al., 1998). These amyloid aggregates cause cellular toxicity, inflammation, and eventually cell death, which all lead to clinical symptoms such as tremor, loss of motor function, and executive dysfunction (Sharon et al., 2003). Due to our lack of understanding of PD mechanisms and the function of α-synuclein, it is critical for both scientists and clinicians to better characterize α-Synuclein aggregation and be able to identify α-Synuclein aggregates in the brain. Historically, like other amyloid aggregates implicated in NDs such as tau or Aβ, α-synuclein can be detected by small fluorescent molecules such as Thioflavin-T (ThT) and Congo Red (CR) (Naiki et al., 1989; LeVine, 1999; Campioni et al., 2010; Fernandez-Flores, 2011). Recent studies showed the importance of oligomeric species, as opposed to fibrils, in PD progression and their role in detrimental neuronal death. PD oligomers are shown to damage synaptic functions in dopaminergic neurons, causing calcium influx, and neuronal death in both cell culture and mouse models (Danzer et al., 2007; Bengoa-Vergniory et al., 2017). Despite the biological and clinical significance of α-Synuclein oligomers, many conventional probes cannot detect α-Synuclein oligomeric aggregates with high specificity, (Ingelsson, 2016; Bengoa-Vergniory et al., 2017), and limited progress has been made in this endeavor. α-Synuclein oligomers are structurally heterogeneous, and no high-resolution structure is available, hindering rational design of oligomer-specific probes. Many α-Synuclein oligomers share β-sheet-rich secondary structure with fibrils, resulting in poor discrimination of different aggregate species (Celej et al., 2008; Ghosh et al., 2015; Pieri et al., 2016; Yoo et al., 2022). In this section, we summarize recent developments of fluorescent probes that are reported to be able to detect α-Synuclein oligomers (Figure 10).

### 4.1 The development of fluorescent probes that target α-synuclein oligomers

Numerous probes have been developed to detect α-synuclein deposits in tissue (Xu et al., 2020; Haque and Maity, 2023). Although most of these probes show improved binding affinity for α-Synuclein compared to the micromolar affinity of ThT, they do not distinguish the different aggregation states among α-Synuclein species (e.g., monomer vs. oligomer vs. fibril). Despite the lack of aggregation specificity, these probes remain valuable as the foundation for the development of future α-Synuclein oligomer-specific probes. For more information regarding α-Synuclein probe development in general, we refer readers to other reviews that summarize reported α-Synuclein binding fluorescent probes (Xu et al., 2020; Haque and Maity, 2023). In this section, we will review the recent α-Synuclein fluorescent probe development, with a highlight on oligomer-binding probes.

### 4.2 State-specific α-synuclein detection probes

Due to recent revelations about α-Synuclein oligomer toxicity and aforementioned difficulties in developing oligomer-specific α-Synuclein probes, only a few papers in recent years have reported on probes that bind to α-Synuclein oligomers. Most of these probes are phrased as aggregation monitoring probes, which means they bind to diverse α-Synuclein aggregate species as their photophysical properties change during the monomer to fibril aggregation process. One such example was developed by the Jovin group where they screened derivatives of the hydrophobicity-sensitive fluorescent probe N-arylaminonaphthalene sulfonate (NAS). (Celej et al., 2008). As α-Synuclein aggregates, the polarity of binding sites putatively changes; therefore, a polarity-sensitive probe can monitor changes in aggregation states. By incubating NAS derivatives with monomeric α-Synuclein, the researchers found several molecules that exhibited fluorescence enhancement during aggregation. One such molecule, **bis-TNS** (Figure 10), also showed a shift in its emission. Other photophysical properties were also measured, and they found two molecules, **bis-TNS** and **bis-ANS**, that report changes in fluorescence lifetime (τ) as the aggregation process proceeds. The researchers claim that such changes in emission wavelength and τ could be used to show different aggregation states and binding to diverse α-Synuclein aggregation species. The Jovin group further explored probes that have different properties during aggregation and developed a 3-hydroxychromones (3HC) probe **AS140-MFC** (Yushchenko et al., 2010). **AS140-MFC** is prepared by introducing sensor molecules with covalent adducts of Ala-to-Cys mutants of α-Synuclein with a thiol-reactive maleimide probe (**MFC**) (Figure 10). **AS140-MFC** probe has two excited states, and when incubated with α-Synuclein, the ratio of these two states changes due to polarity differences which reflect the transition of aggregation states. A similar approach for finding α-Synuclein oligomer probes through enhancing hydrophobic interactions has been adopted by other groups as well. The Yarmoluk group published a series of tri- and pentamethine cyanine probes with bulky phenol and alkyl groups and tested binding with different α-Synuclein aggregates in solution (Kovalska et al., 2012). Of the reported probes, **SL-631** and **SH-299** (Figure 10) showed strong fluorescence enhancement when bound to α-Synuclein oligomers and less enhancement for fibrils in solution. Despite these two probes showing promise for detection of α-Synuclein oligomers, it remains to be seen if they can detect α-Synuclein oligomers specifically in tissue.

A common strategy to develop fluorescent probes for protein aggregates is aggregation-induced emission (AIE). These molecules show weak fluorescence when unbound in solution but become fluorescent upon interacting with targets (Hong et al., 2009; Z; Zhao et al., 2020). Tetraphenylethene tethered with triphenylphosphonium (**TPE-TPP**, Figure 10) was designed based on known π-stacking and hydrophobic interactions between ThT and higher-ordered amyloid aggregates. **TPE-TPP** was incubated during α-Synuclein aggregation and showed fluorescence enhancement at earlier time points compared to ThT, indicating potential binding with α-Synuclein oligomers. Comparison with immunoblotting of **TPE-TPP** with an α-Synuclein oligomer antibody also confirmed oligomer binding. This probe was later tested with other aggregated proteins derived from α-lactalbumin, κ-casein, and hen egg white lysozyme (HEWL), showing potential to be an aggregation monitoring probe for various amyloidogenic proteins in addition to α-Synuclein (Kumar et al., 2017).

Since ThT is a traditional amyloid dye to detect aggregates, researchers have used it as inspiration for developing oligomer-specific probes. One such ThT derivative, **ThX**, was shown to bind to α-Synuclein oligomers by showing early fluorescence enhancement during aggregation of α-Synuclein in solution. (Yap et al., 2011). Further studies suggested **ThX** might bind to heterogeneous α-Synuclein oligomers not detected by ThT.

Few reports have been made towards detection of α-Synuclein oligomers in biologically relevant conditions such as tissue or cell culture. Inspired by GFP, a widely used genetically encoded fluorescence protein, the Zhang group created probes based on GFP’s fluorescent chromophore 4-hydroxybenzylidene-imidazolinone (**HBI**, Figure 10). (Liu et al., 2018) The Zhang lab synthesized derivatives of **HBI** to improve aggregated protein detection by increasing π conjugation, restricting bond rotation, and substituting the phenol group with electron-donating substituents. The probes were incubated in solution with α-Synuclein and fluorescence intensity increase was detected early in the aggregation process, which the authors suggest is due to binding to α-Synuclein oligomers. The authors then incubated the **HBI** derivative probes in cell culture and visualized Huntingtin exon 1 protein (Htt) and superoxide dismutase 1 (SOD1) aggregation *in vivo*. Although the ability of **HBI** derivatives to detect α-Synuclein was not tested in cell culture, this work showed the potential of fluorescent probes to visualize and study the function of oligomers in a living system.

### 4.3 Summary of current efforts to develop α-synuclein oligomeric fluorescent probes

The development of fluorescent probes for oligomeric α-Synuclein has gained attention from many researchers in recent years as studies show that α-Synuclein oligomers are more toxic to neurons and are important in the pathology of many synucleinopathies. Currently, most design routes are based on existing probes for higher-ordered α-Synuclein aggregates, and the focus has been to monitor the α-Synuclein aggregation process rather than an oligomeric-specific probe. These aggregation-monitoring probes bind to both oligomeric and higher-ordered α-Synuclein aggregates but can also respond earlier during the aggregation process or show slightly different photophysical properties when bound to oligomers versus fibrils in solution. These probes have advanced our understanding of α-Synuclein oligomers, but they still lack binding specificity and significant contrast between different states of α-Synuclein aggregate species. Furthermore, most of the probes reported have only been tested with *in vitro* assays with minimal validation of the presence or characterization of α-Synuclein oligomers in these experiments.

Moving forward, many directions can be taken for developing α-Synuclein oligomer-specific probes. Chemical modifications to probes that detect higher-ordered α-Synuclein aggregates represents an actionable starting point. Furthermore, chemical modifications in tandem with computer-aided design and pre-screening of fluorescent probes can further advance the field. While models for α-Synuclein oligomers do not currently exist, structures of α-Synuclein fibrils have been reported and may allow for development of rational design principles for targeting α-Synuclein in general (Guerrero-Ferreira et al., 2018). In addition, hydrophobicity differences between various aggregation states of α-Synuclein may allow for new design principles for chemical probes that specifically detect α-Synuclein oligomers (Lee et al., 2018).

## 5 Conclusion

Developing fluorescent probes that can selectively detect oligomers of amyloidogenic proteins has proven to be a challenging yet exciting task for chemists to pursue. With numerous studies that suggest increased pathological activity of oligomers over higher-ordered aggregates in many NDs, there has been a recent shift in the focus of many research laboratories towards developing fluorophores that can detect these oligomeric species across different NDs. While rational design principles for oligomers are not fully established and more robust probes with improved biological properties are still needed, we have summarized probes that have been reported to bind to oligomers in literature and discussed potential design guidelines based on literature precedence. Towards the development of fluorescent probes that bind to Aβ oligomers, the pre-screening of fluorescent probes that can bind to a trimeric structural model for Aβ (PDB 4NTR) may lead to novel probes that target oligomers over fibrils. In addition, the use of increased steric bulk on the probe has shown to increase interaction with the cavity of Aβ oligomers over fibrils and allows for improved specificity over fibrils. While exciting advances have been made towards the development of fluorescent probes that can specifically target Aβ oligomers, the development of probes that can detect phosphorylated tau oligomers is more limited. Some research groups have shown that using a Zn^2+^ recognition motif and chemical modifications of existing tau probes can lead to the recognition of phosphorylated tau in solution studies. However, many of these tau probes have yet to be evaluated in biologically relevant samples such as brain tissue or biological fluids such as cerebrospinal fluid (CSF). Elevated levels of soluble aggregates of amyloidogenic proteins in CSF have been reported in patients with AD, therefore the detection of endogenous levels of these aggregates in CSF may complement AD diagnosis as a less invasive avenue that can potentially allow for monitoring of AD progression (Blennow, 2004; De et al., 2019; Povala et al., 2021). The design of fluorescent probes that can detect oligomeric α-Synuclein are currently based on scaffolds that showed promise as a probe for detecting Lewy bodies and neurites and have been limited to experiments *in vitro.* In analogy to the promising work done with targeting Aβ oligomers, the pre-screening of fluorescent probes that can bind to oligomeric models of tau and α-Synuclein *in silico* may provide a starting point for developing fluorescent probes that can bind to phosphorylated tau oligomers and oligomeric α-Synuclein. While models for higher-ordered aggregates of tau and α-Synuclein exist and were identified using various techniques such as x-ray crystallography, nuclear magnetic resonance, and cryo-electron microscopy, there is an urgent need for high-resolution structures of tau and α-Synuclein oligomers. Molecular docking studies to a published trimeric structure of synthetic Aβ played an instrumental role in the rational design of several probes for Aβ oligomers, and analogous methods may prove useful for developing probes that can detect tau and α-Synuclein oligomers if reliable structures are available. However, as mentioned earlier, aggregated synthetic and recombinant proteins can produce structures that are different from patient-derived samples. Furthermore, due to the lack of standardized protocols for aggregation it has been reported that different structures can arise depending on several factors including choice of added reagents and procedures for aggregation (Zhang et al., 2019; Al-Azzani et al., 2022). Therefore, if possible, the use of structures obtained from patient-derived samples for docking studies may serve as a better predictive model for the development of oligomer binding probes. Lastly, more rigorous, and unambiguous assays that confirm the aggregation state of amyloidogenic proteins in solution using common techniques such as TEM, Western blot, gel-staining, and the verification in cells/tissue with appropriate antibodies would improve the standard in the field and pave the way for the development of improved fluorescent probes that are specific to oligomers. Furthermore, the development of fluorescent probes that can detect oligomers for *in vivo* imaging in pre-clinical animal models for NDs could serve as valuable proof-of-concept for future *in vivo* imaging of oligomeric aggregates in humans. We hope that this review will engage a wide range of chemists who are enthusiastic about developing oligomer-specific fluorescent probes for NDs that may aid in the earlier diagnosis of amyloid-associated diseases.
